# Exosomes Engineered to Express a Cardiomyocyte Binding Peptide Demonstrate Improved Cardiac Retention *in Vivo*

**DOI:** 10.1038/s41598-019-46407-1

**Published:** 2019-07-11

**Authors:** Kyle I. Mentkowski, Jennifer K. Lang

**Affiliations:** 1 Department of Medicine, Division of Cardiology, Jacobs School of Medicine and Biomedical Sciences, Buffalo, N.Y. 14203 United States of America; 2 Department of Biomedical Engineering, University at Buffalo, Buffalo, N.Y. 14260 United States of America; 3 VA WNY Healthcare System, Buffalo, N.Y. 14215 United States of America

**Keywords:** Molecular engineering, Cell signalling, Heart stem cells, Cardiac regeneration

## Abstract

Injury to the heart results in cardiomyocyte cell death and can lead to pathological remodeling of remaining cells, contributing to heart failure. Despite the therapeutic potential of new drugs and small molecules, there remains a gap in the ability to efficiently deliver cardioprotective agents in a cell specific manner while minimizing nonspecific delivery to other organs. Exosomes derived from cardiosphere-derived cells (CDCs) have been shown to stimulate angiogenesis, induce endogenous cardiomyocyte proliferation and modulate cardiomyocyte apoptosis and hypertrophy. While innately cardioprotective at high doses, unmodified CDC-exosomes demonstrate limited cardiac tropism. To generate an efficient exosomal delivery system that can target cardiomyocytes, we engineered CDCs to express Lamp2b, an exosomal membrane protein, fused to a cardiomyocyte specific peptide (CMP), WLSEAGPVVTVRALRGTGSW. Exosomes isolated from engineered CDCs expressed CMP on their surface and retained their native physical properties. Targeted exosomes resulted in increased uptake by cardiomyocytes, decreased cardiomyocyte apoptosis, and higher cardiac retention following intramyocardial injection when compared with non-targeted exosomes. Importantly, we established a novel targeting system to improve exosomal uptake by cardiomyocytes and laid the foundation for cell-specific exosomal delivery of drug and gene therapies to improve the functional capacity of the heart following both ischemic and non-ischemic injury.

## Introduction

Cardiovascular disease (CVD) continues to be a leading and growing cause of morbidity and mortality worldwide^1^. Despite major advancements in management, heart disease remains a progressive condition. Once cardiomyocytes are damaged, they cannot be replaced efficiently, leading to a decline in heart function. Following a functionally significant myocardial infarction, an estimated one billion cardiomyocytes are lost in a wave of cellular necrosis that sweeps from the subendocardium to the subepicardium. The remaining cardiomyocytes in the remote and border zone can become overloaded and hypertrophied, contributing to eventual pathological remodeling and heart failure. In addition to ischemia induced cellular necrosis, cardiomyocytes also die in bulk by apoptosis and autophagy in heart failure, ischemia-reperfusion, and non-ischemic cardiomyopathies^2^. Despite a myriad of pre-clinical and early stage clinical trials, the ability of transplanted stem cells to engraft in any great number in host myocardium and differentiate into functional adult cardiomyocytes with integrated electro-mechanical couplings is limited^3^. As such, the ability to target cardioprotective therapy to this at-risk endogenous population represents an attractive means of modulating the subsequent cardiac remodeling that can occur following ischemic and non-ischemic injury to the heart.

Exosomes, a subset of extracellular vesicles (EVs) formed during the invagination of multivesicular bodies (MVBs), have more recently been recognized as therapeutic delivery vehicles. Secreted by all cells in both physiological and pathological conditions, these nanosized carriers shuttle cell-specific lipids, nucleic acids and proteins throughout the circulatory system. Various stem cell-derived exosomes demonstrate cardiovascular bioactivity, posing them as a promising means of acellular therapy for heart disease^4^. Exosomes from cardiosphere-derived cells (CDCs) have been shown to recapitulate the therapeutic effects of CDCs in animal models of heart disease^5,6^. Containing an array of cardioprotective cargo, they have been shown to stimulate angiogenesis, induce endogenous cardiomyocyte proliferation and modulate cardiomyocyte apoptosis and hypertrophy^7,8^. When injected in animal models of ischemia-reperfusion, CDC-derived exosomes reduce infarct size and improve left ventricular function.

Despite an observed efficacy at purified and concentrated doses, the therapeutic potential of exosomes is largely limited by their biodistribution^4^. With few exceptions^9^, most naturally secreted exosomes demonstrate limited tropism to a specific cell type. In this article, we sought to enhance cardiomyocyte endocytosis of cardiosphere-derived cell exosomes through controlled expression of a cardiomyocyte targeting peptide (CMP) on their exosomal surface. To achieve this aim, we ligated a cardiomyocyte specific peptide^10^ to the extra-exosomal N-terminus of Lamp2b. Cardiomyocyte-targeted CDC exosomes demonstrate improved uptake into cardiomyocytes *in vitro*, decreased cardiomyocyte apoptosis, and enhanced cardiac retention *in vivo*, highlighting the specificity and ability of this delivery system to target a cell population of interest.

## Results

### Unmodified exosomes from cardiosphere-derived cells do not demonstrate cardiac tropism

**Figure 1:**
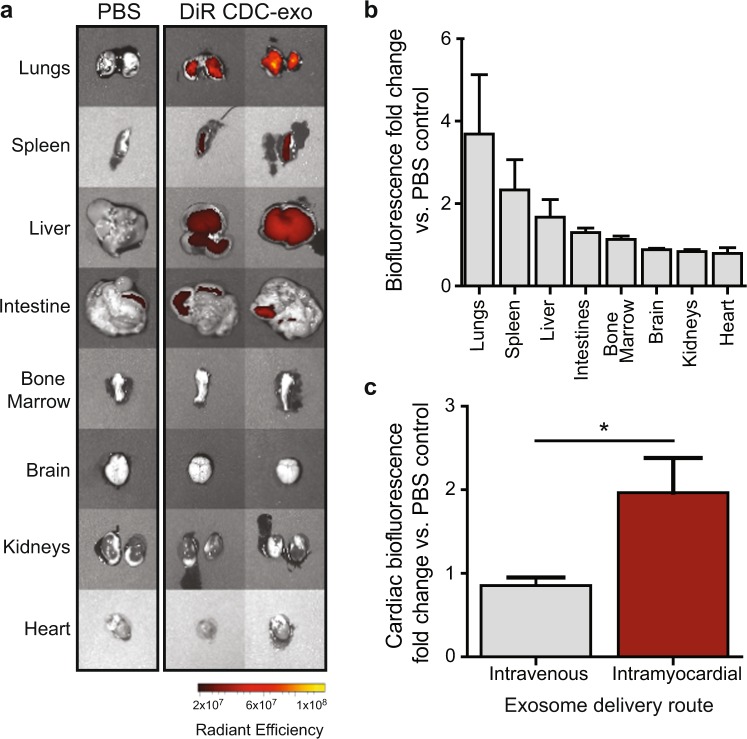
CDC-exosome biodistribution varies by route of administration. Human CDC-exosomes were labeled with 1 μM DiR and residual free dye removed. C57BL/6 mice received either an intravenous (retro-orbital) or intramyocardial injection of either exosomes or PBS vehicle. **(a)** Representative IVIS images of organs 2 hours post intravenous injection of exosomes or PBS control demonstrate highest uptake in the lungs, spleen and liver. **(b)** Normalized biofluorescence signal in each organ following intravenous exosome delivery at 2 hours, expressed as a ratio of DiR CDC-exosomes/PBS control (n = 3). **(c)** Normalized cardiac biofluorescence in animals receiving either intravenous or intramyocardial exosome injections at 2 hours, expressed as a ratio of DiR CDC-exosomes/PBS control. Intramyocardial injections of DiR-labeled CDC-exosomes demonstrates increased cardiac retention at 2 hours compared with intravenous injection (n = 5–6 mice per group). p < 0.05 using two-tailed Student’s t-test. All data expressed as mean ± SEM.

To assess whether cardiosphere-derived cells produce exosomes with inherent cardiac tropism, we studied the *in vivo* biodistribution of unmodified CDC-derived exosomes following systemic injection in wild type non-infarcted mice. Mice received retro-orbital injections of either DiR labeled CDC-exo or PBS followed by *ex vivo* whole-organ fluorescence imaging to assess the relative concentration of labeled exosomes. At 2 hours post systemic injection, DiR labeled non-modified CDC-exosomes accumulated primarily in lung, spleen and liver, with lower levels in the intestines and bone marrow, and nearly non-detectable levels in the brain, kidneys and heart (Fig. 1a,b).

To determine if the route of exosome administration influenced exosome biodistribution, we compared the *in vivo* biodistribution of systemic and intramyocardial injected unmodified exosomes into wild type non-infarcted mice. Mice received retro-orbital or intramyocardial injections of either DiR labeled CDC-exo or PBS followed by *ex vivo* cardiac fluorescence imaging 2 hours post-delivery. As compared with mice that received a systemic intravenous injection of exosomes, non-infarcted wild type mice that received intramyocardial injections of CDC-exosomes exhibited a modest enhancement in cardiac retention (2.0 ± 0.4 vs. 0.9 ± 0.1) as measured by biofluorescence fold change compared to PBS control (n = 5–6 mice per group, mean ± SEM; p < 0.05 using two-tailed t-test) (Fig. 1c).

### Indirect engineering of cardiosphere-derived cells modifies CDC-derived exosomes to express a cardiomyocyte specific binding peptide on their surface

**Figure 2:**
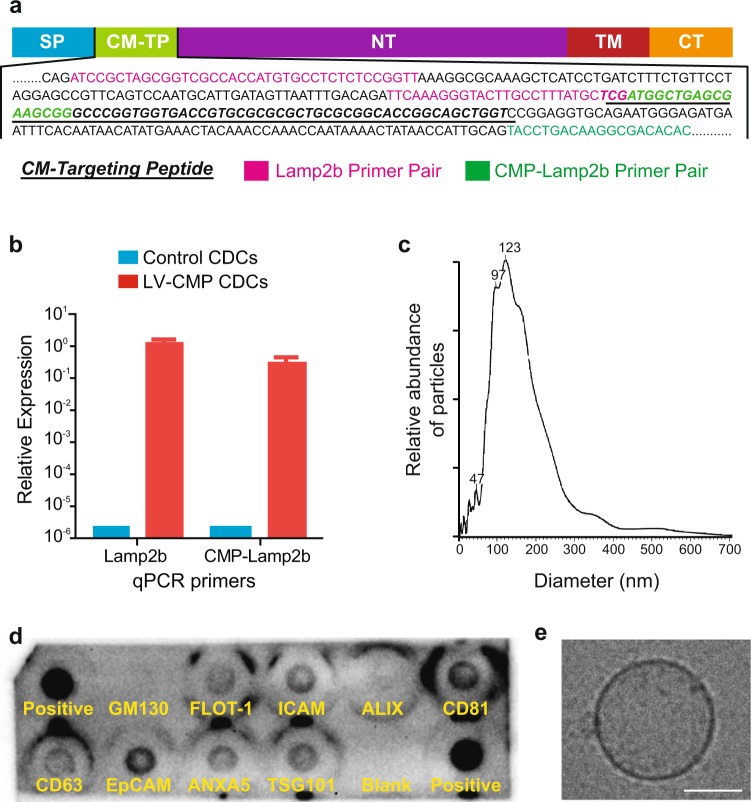
Characterization of engineered CDCs and CMP-targeted exosomes. (**a**) Schematic representation of the modified Lamp2b protein. Primer pairs for qPCR are highlighted, as well as the location of relevant primer pairs and the cloned cardiomyocyte targeting peptide. SP: Signal Peptide, CM-TP: Cardiomyocyte Targeting Peptide, NT: N-Terminus, TM: Trans-membrane Region, CT: C-terminus. (**b**) qPCR of control CDCs or CDCs transduced with LV-CMP with primers specific for Lamp2b or overlapping CMP and Lamp2b. (**c**) CMP-targeted exosome size distribution as determined by Nanoparticle Tracking Analysis. (**d**) Exo-Check Exosome Antibody Array for exosomal surface protein marker detection and assessment of cellular contamination. (**e**) Cryo-TEM image of CMP exosome isolated from serum free tissue culture. Scale bar: 50 μm.

To enhance cardiac tropism, we conferred cardiomyocyte targeting capabilities to CDC-derived exosomes using a strategy of indirect cellular engineering. Modifying a previously characterized protocol for displaying epitopes on the external exosome surface^11^, we fused a cardiomyocyte-specific binding peptide (WLSEAGPVVTVRALRGTGSW)^10^ to the extra-exosomal N-terminus of murine transmembrane protein Lamp2b (Fig. 2a). The selected cardiomyocyte binding peptide contains a 12 amino acid segment with sequence homology to a peptide in tenascin-X, an extracellular matrix protein, in mice (BAA24436.1), pigs (NP_001116676.1) and humans (AAB47488.1). We verified proper ligation and expression of CMP with Lamp2b by RT-PCR of CDCs followed by Sanger sequencing (Supplementary Fig. S5). When this fusion cassette was expressed in cardiosphere-derived cells using a lentiviral vector, transduced CDCs generated exosomes expressing the peptide of interest on their surface. Expression of CMP-Lamp2b construct in CDCs was further confirmed by quantitative PCR analysis of transduced cardiosphere-derived cells (Fig. 2b). These modifications did not affect the physical properties of CDC-derived exosomes (previously characterized in^7^) as nanoparticle tracking analysis of CMP exosomes showed a typical size distribution (mean diameter, 123 nm) (Fig. 2c) and cryo-TEM demonstrated the expected exosomal morphology of small, round vesicles with a clearly discernible lipid bilayer (Fig. 2e). CMP exosomes were positive for known exosomal markers CD63, CD81, ALIX, FLOT1, ICAM1, EpCam, ANXA5 and TSG101 with preparations negative for any cellular contamination (by cis-Golgi marker GM130) (Fig. 2d). In addition, surface particle charge analysis demonstrated an expected negative zeta potential (−35 mV) (Supplementary Fig. S6).

### CDC-derived exosomes expressing CMP confer enhanced cardiomyocyte-specific exosomal uptake *in vitro*

**Figure 3:**
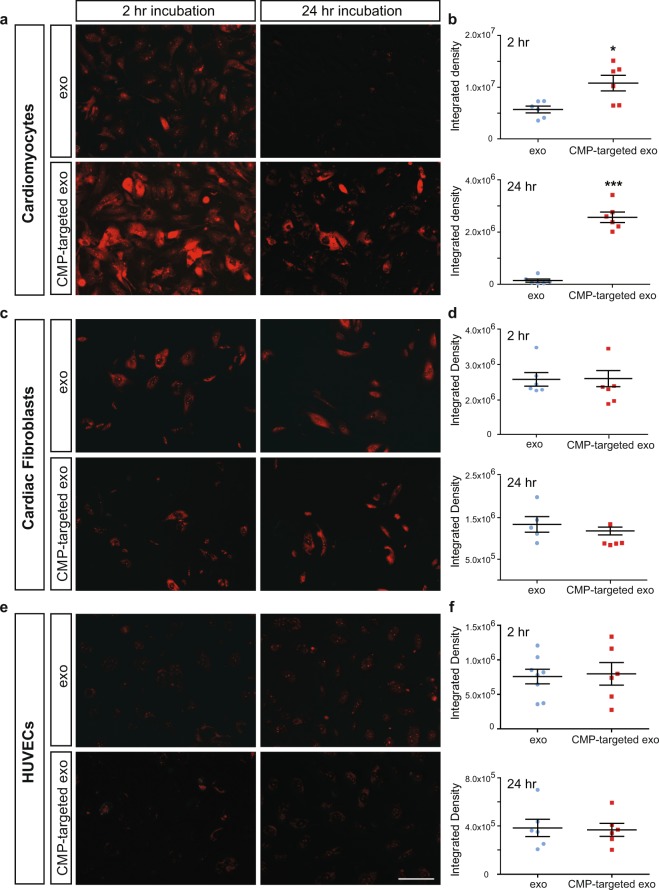
Cardiomyocytes demonstrate enhanced uptake of CMP-targeted exosomes *in vitro*. CDC derived control and CMP-targeted exosomes labeled with Exo-Red were incubated with (**a**,**b**) primary neonatal mouse cardiomyocytes, (**c**,**d**) primary adult human cardiac fibroblasts, and (**e**,**f**) human umbilical vein endothelial cells (HUVECs) and imaged at 2 and 24 hours to analyze cellular uptake. (**a**,**b**) Cardiomyocytes exhibited increased uptake of CMP-targeted exosomes at 2 and 24 hours when compared with cardiomyocytes incubated with non-modified exosomes (n = 6). *p < 0.01, ***p < 0.0001 using a two-tailed Student’s t-test. Unlike cardiomyocytes, neither (**c**,**d**) cardiac fibroblasts nor (**e**,**f**) HUVECs demonstrated any statistically significant difference in exosome uptake between control CDC exosomes and their targeted counterparts at 2 or 24 hours (n = 6). Scale bar: 200 μm. p > 0.05 using a two-tailed Student’s t-test. Data represented as mean ± SEM.

We performed an *in vitro* uptake assay to determine if CMP-targeted CDC-derived exosomes were more readily endocytosed by cardiomyocytes than non-targeted exosomes. Primary neonatal mouse cardiomyocytes (mCMs) were incubated with exo-Red labeled unmodified CDC-derived exosomes (exo) or CMP-targeted exosomes and imaged at 2 and 24 hours. At 2 hours, cardiomyocytes exhibited increased uptake of CMP-targeted exosomes (1.1 × 10^7^ ± 1.5 × 10^6^ integrated density) by two-fold when compared with non-targeted exosomes (5.7 × 10^6^ ± 6.5 × 10^5^ integrated density) as assessed by fluorescence integrated density (n = 6, mean ± SEM, p = 0.011, t-test). While total fluorescence was lower at 24 hours, the ratio of fluorescence signal increased to a difference of 18-fold between CMP-targeted exosomes and unmodified exosomes (2.6 × 10^6^ ± 2.0 × 10^5^ vs. 1.4 × 10^5^ ± 6.1 × 10^4^ integrated density, p < 0.0001, t-test) (Fig. 3a,b).

To determine if increased uptake of CMP-targeted exosomes was specific to cardiomyocytes, we assessed exosome uptake by other cardiac cell populations. We found no significant difference in exosomal uptake at 2 or 24 hours between targeted and non-targeted exosomes by human primary adult cardiac fibroblasts (2 hr: 2.6 × 10^6^ ± 1.9 × 10^5^ vs. 2.6 × 10^6^ ± 2.3 × 10^5^; 24 hr: 1.3 × 10^6^ ± 1.9 × 10^5^ vs. 1.2 × 10^6^ ± 9.4 × 10^4^) (Fig. 3c,d) or HUVECs (2 hr: 7.6 × 10^5^ ± 1.0 × 10^5^ vs 8.0 × 10^5^ ± 1.6 × 10^5^; 24 hr: 3.8 × 10^5^ ± 7.1 × 10^4^ vs. 3.7 × 10^5^ ± 5.3 × 10^4^) (Fig. 3e,f) (n = 6, p > 0.05, t-test).

**Figure 4:**
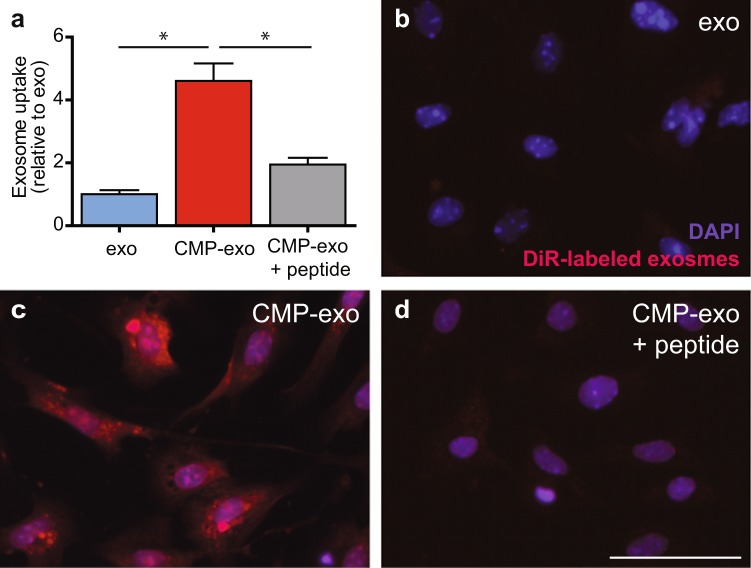
Synthetic 20-mer peptide blocks cardiomyocyte uptake of CMP-targeted exosomes. Freshly isolated primary neonatal mouse cardiomyocytes were assayed for uptake of Exo-Red labeled CMP-targeted exosomes in the presence or absence of the synthetic peptide, WLSEAGPVVTVRALRGTGSW. **(a)** Graph depicts the fold change in exosome uptake of targeted-exosomes +/− peptide relative to non-targeted exo (set to 1) at 2hrs. **(c)** Cardiomyocytes showed enhanced uptake of CMP-targeted exosomes (4.6 ± 0.6 vs. 1 ± 0.1). (**d**) Targeted exosome uptake was reduced in the presence of 1 μM of synthetic peptide (4.6 ± 0.6 to 1.94 ± 0.2) and showed no significant difference to (**b**) non-targeted exosome uptake (n = 3). Scale bar: 200 µm. *p < 0.05 using a one-way ANOVA followed by Tukey’s Multiple Comparison post-hoc test. Data are represented as mean ± SEM.

To further explore if enhanced cardiomyocyte uptake of CMP-targeted exosomes was specific to the CMP peptide in our cloned fusion protein, we assayed freshly isolated primary neonatal mouse cardiomyocytes for uptake of Exo-Red labeled CMP-targeted exosomes in the presence or absence of 1 μM of the synthetic peptide, WLSEAGPVVTVRALRGTGSW. As previously shown, cardiomyocytes demonstrated enhanced uptake of CMP-targeted exosomes relative to non-targeted exosomes (fold change of 4.6 ± 0.6). However, when 1 μM of synthetic peptide was added to the cardiomyocytes prior to the addition of CMP-targeted exosomes, exosomal uptake was reduced by 2.7 fold (4.6 ± 0.6 to 1.9 ± 0.2) and showed no significant difference when compared with cardiomyocyte uptake of non-targeted exosomes (n = 3, p < 0.05, one-way ANOVA followed by Tukey’s Multiple Comparison post-hoc test) (Fig. 4a–d).

### CMP-targeted exosomes further reduce CDC-exosome mediated cardiomyocyte apoptosis *in vitro*

**Figure 5:**
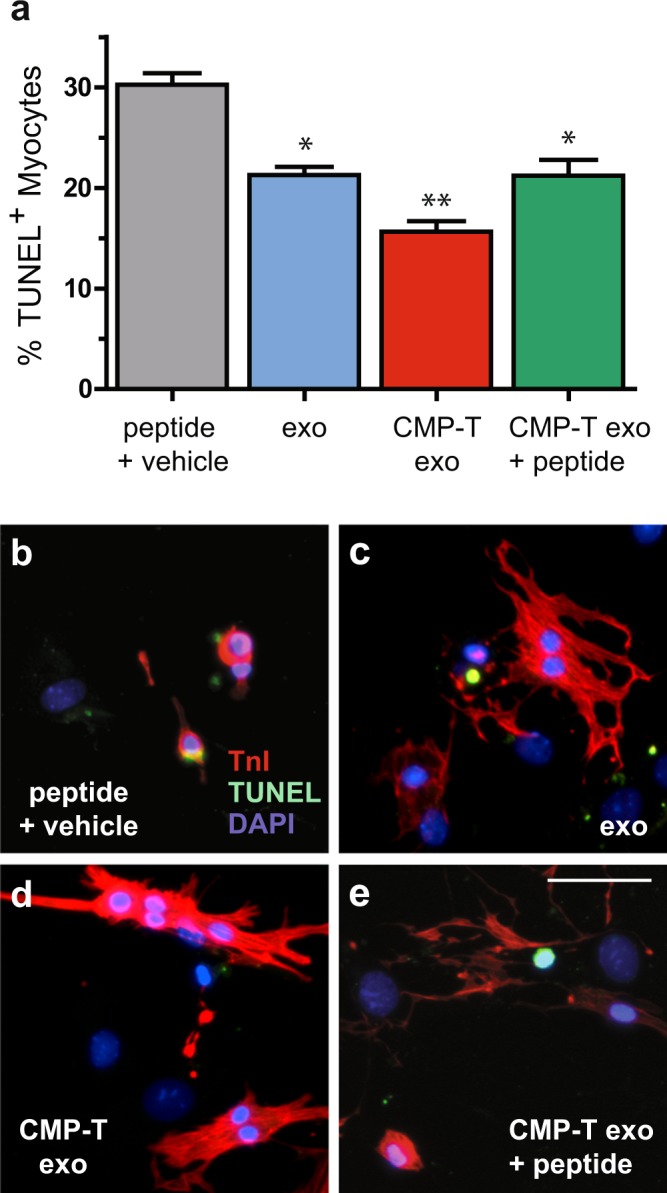
CMP-targeted exosomes significantly decrease cardiomyocyte programed cell death *in vitro* at one week in culture. Primary mouse neonatal cardiomyocytes were treated with either synthetic peptide, WLSEAGPVVTVRALRGTGSW, plus vehicle control (10 µM DMSO), control exosomes (exo), CMP-targeted exosomes, or CMP-targeted exosomes + synthetic peptide for 24 hours and assessed for myocyte apoptosis. (**a**,**d**) Cardiomyocytes treated with CMP-targeted exosomes exhibited fewer terminal deoxynucleotidyl transferase nick end labeling (TUNEL)-positive nuclei at one week when compared with **(a,c)** control exosomes. **(a**–**e)** The enhanced anti-apoptotic effect of targeted vs. control exosomes when compared with the peptide plus vehicle control was abrogated when cardiomyocytes were co-treated with **(e)** CMP-targeted exosomes and synthetic peptide. Scale bar: 100 µm. *p < 0.05 compared with peptide plus vehicle control using a one-way ANOVA followed by Tukey’s Multiple Comparison post-hoc test. **p < 0.05 compared with exo and CMP-targeted exo + peptide using a one-way ANOVA followed by Tukey’s Multiple Comparison post-hoc test. Data are represented as mean ± SEM.

To investigate whether enhanced cardiomyocyte uptake *in vitro* translated to reduced myocyte cell death by apoptosis, we incubated mCMs with our peptide plus vehicle control, unmodified CDC-exosomes, and CMP-targeted CDC-exosomes in the presence or absence of 1 μM of the CMP synthetic peptide. Consistent with our prior published results^7^, control CDC-exosomes had a significant effect on preventing myocyte cell death from apoptosis. mCMs treated with CDC-exos exhibited fewer terminal deoxynucleotidyl transferase nick end labeling (TUNEL)-positive nuclei at one week (21.27% ± 0.81%) when compared with cardiomyocytes treated with peptide plus vehicle control alone (30.25% ± 1.15%) (mean ± SEM, n = 3, p < 0.0001 using one-way ANOVA with Turkey’s post hoc test). Interestingly, when compared with levels of apoptosis seen with control exosomes at one week (21.27% ± 0.81%), treatment with CMP-targeted exosomes demonstrated further reduction in cardiomyocyte apoptosis (15.63% ± 1.06%) (mean ± SEM, n = 3, p < 0.05 using one-way ANOVA with Turkey’s post hoc test). This additive effect was abrogated in the presence of the CMP synthetic peptide (21.23% ± 1.58%) (mean ± SEM, n = 3, ns) demonstrating specificity of reduced mCM apoptosis to the CMP peptide (Fig. 5a,c–e).

### CMP-targeted CDC-derived exosomes demonstrate cardiac tropism following intramyocardial injection

**Figure 6:**
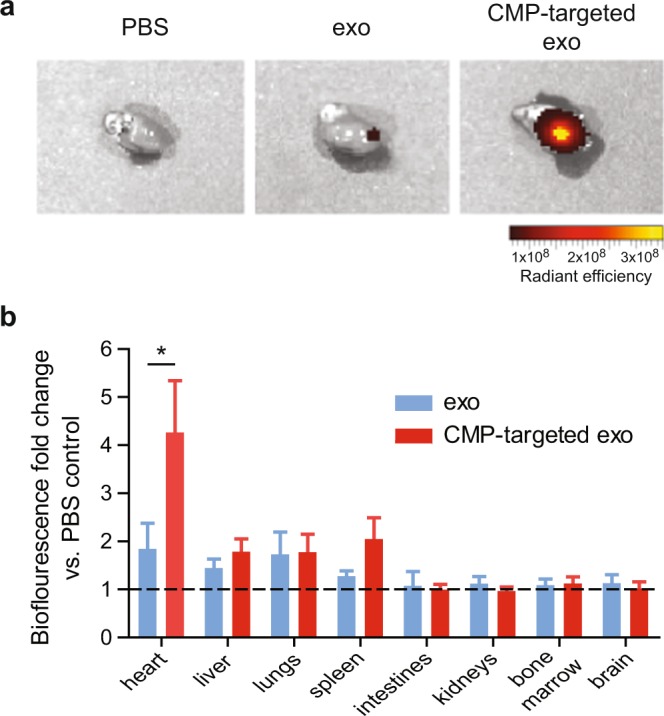
CMP-targeted exosomes demonstrate increased cardiac retention 2 hours after intramyocardial injection *in vivo*. C57BL/6 mice received an intramyocardial injection of either DiR-labeled control exosomes, CMP-targeted exosomes, or PBS vehicle. **(a)** Representative IVIS images of hearts 2 hours post intramyocardial injections demonstrate enhanced uptake of CMP-targeted exosomes vs PBS and control exosomes. **(b)** Biofluorescence was measured in each organ of mice receiving intramyocardial delivery of PBS, control exosomes or CMP-targeted exosomes at 2 hours. Graph depicts the fold change in biofluorescence of intramyocardial delivered control exosomes (exo) vs. CMP-targeted exosomes, demonstrating increased retention of CMP-targeted exosomes in the heart (4.3 ± 1.0 vs. 1.9 ± 0.5) compared with their PBS control (n = 5–7 mice per group). *p < 0.001 using a two-way ANOVA followed by Bonferroni post-hoc test. Data are represented as mean ± SEM.

Finally, to test if the specificity of improved cardiomyocyte uptake of CMP-targeted exosomes *in vitro* translated to enhanced cardiac tropism *in vivo*, we performed blinded intramyocardial injections of DiR-labeled exosomes followed by *ex vivo* whole-organ fluorescence imaging at 2 hours post-injection (n = 5–7 mice per group; CMP-targeted exosomes, unmodified exosomes, PBS). *Ex-vivo* imaging analysis revealed improved retention of CMP-targeted CDC-exosomes in the heart by a fold change of 4.3 ± 1.0 vs. control CDC-exosomes (1.9 ± 0.5) when compared with PBS control (mean fold change ± SEM, p < 0.001 using two-tailed ANOVA with Bonferroni post hoc test) (Fig. 6a,b). No statistically significant difference was found in any other organ when comparing CMP-targeted CDC-exosomes to their non-engineered CDC-exosome control (p > 0.05 using two-tailed ANOVA with Bonferroni post hoc test) (Fig. 6b).

## Discussion

The biodistribution of exosomes influences their therapeutic efficacy as well as their off-site effects^4^. To improve exosome targeting and delivery of cardioprotective cargo to cardiomyocytes, we engineered human cardiosphere-derived cells to generate exosomes expressing a cardiomyocyte binding peptide on their surface. This surface modification improved exosomal uptake by cardiomyocytes *in vitro*, reduced cardiomyocyte apoptosis, and increased exosome cardiac retention *in vivo*.

With few exceptions, most naturally secreted exosomes demonstrate limited tropism to a specific cell type^4^. In the absence of ischemic injury, we found that systemically administered CDC-derived exosomes do not preferentially target the heart, but instead localize mainly to the spleen, liver and lungs (Fig. 1a,b). This is consistent with numerous studies demonstrating that intravenously injected exosomes are rapidly cleared by macrophages of the mononuclear phagocyte system (MPS) and preferentially accumulate in MPS tissues such as the liver, spleen and lungs^12,13^. Clearance of exosomes in macrophage depleted mice is significantly delayed compared to control animals, suggesting that macrophages play a pivotal role in the clearance of EVs from blood circulation irrespective of the EV cell of origin^14^. This appears to be mediated by recognition of the negatively charged phosphatidylserine on the surface of exosomes through the class A macrophage scavenger receptor^15^. Assessing exosome biodistribution by *ex vivo* organ biofluorescence, we found that unmodified exosomes had the highest accumulation in the lungs two hours after systemic administration (Fig. 1a,b). This was in contrast to previous work that showed high retention in the spleen 24 hours after systemic delivery^4^. This difference in biodistribution pattern may be due to sequestration of CDC-exosomes by circulating macrophages which later migrate to the spleen and liver^16^. In addition to biodistribution of exosomes in MPS tissues, CDC-exosomes also localized to the bone marrow at 2 hours (Fig. 1a,b), highlighting the capability of exosomes to influence activation and recruitment of immune cells post-injury, irrespective of their cardiac localization.

By altering the route of exosome administration, we were able to improve unmodified exosome biodistribution to the heart. When compared with systemic delivery, intramyocardial delivery of non-targeted CDC-derived exosomes resulted in increased cardiac retention at 2 hours (Fig. 1c). Unlike the fenestrated capillaries of the kidneys or the sinusoidal capillaries of the liver and spleen, myocardial capillaries are continuous with tight junctions between their endothelial cells, limiting the movement of macromolecules. As a result, exosomes delivered by a systemic route may have limited access to and a low first pass uptake by the interstitial myocardium. Following ischemic injury, exosomes administered by either an intravenous or intracoronary route may also have difficulty accessing the myocardial interstitium secondary to damaged microvasculature resulting in a “no flow phenomenon”^17^. Consistent with this rationale, prior studies have demonstrated a reduction in scar size and preservation in left ventricular ejection size (LVEF) only when CDC-derived exosomes were delivered via an intramyocardial but not an intracoronary injection. This is supported by our biodistribution data which suggests bypassing systemic delivery for increased cardiac retention.

The proteins expressed on the surface of exosomes are vital in biodistribution and cell targeting. Minor differences in exosomal tetraspanin complexes have been shown to strongly influence target cell selection *in vitro* and *in vivo*^18^. Utilizing this knowledge, we modified prior genetic approaches^11^ to create a fusion cassette that ligated a cardiomyocyte specific peptide with the exosomal transmembrane protein Lamp2b. Alvarez-Erviti *et al*. previously showed that fusion of RVG with Lamp2b enabled exosomes to cross the blood brain barrier and deliver biologically active payloads to neurons^11^. Bellavia *et al*. used a similar model to target Imatinib-loaded exosomes to interleukin 3-receptor positive cells, resulting in inhibition of cancer cell growth *in vitro* and *in vivo*^19^.

To achieve cardiomyocyte targeting capabilities for use in ischemic and non-ischemic models of heart disease, we selected a cardiomyocyte-specific binding peptide (CMP) previously discovered by phage biopanning that was found to bind primary cardiomyocytes 180 times more avidly than control phages^10^. This 20 amino acid peptide (WLSEAGPVVTVRALRGTGSW) has been used to improve cardiomyocyte-specific targeting of liposome delivery systems^20^ and bioreducible polymer delivery systems^21^. For example, a synthesized bioreducible polymer modified with CMP and loaded with Fas siRNA was able to down-regulate Fas gene expression and inhibit cardiomyocyte apoptosis^21^. Use of this peptide also allows for future translation to pre-clinical large animal models, such as the pig, as well as clinical trials in humans, as it targets a region in tenascin-X which is conserved across species. Other cardiac homing peptides have been investigated in liposome models of delivery such as anti-myosin 2G4^22^, anti-cardiac troponin I^23^, and anti-P-selectin^24^. Through phage display biopanning, additional cardiac binding peptides have been identified^25^ with research ongoing to support their efficacy as potential targeting ligands^26,27^.

We demonstrated that CMP-expression on the surface of CDC-derived exosomes did not significantly alter their physical properties of size (Fig. 2c), exosomal surface protein expression (Fig. 2d), morphology (Fig. 2e), and surface charge (Supplementary Fig. S6). *In vitro* uptake analysis showed that both exosome populations were able to be internalized by cardiomyocytes, cardiac fibroblasts, and endothelial cells. In contrast to cardiac fibroblasts and endothelial cells which showed no difference in uptake between CMP-targeted and non-modified exosomes, cardiomyocytes demonstrated a preferential increase in uptake of CMP-targeted exosomes at both 2 and 24 hours (Fig. 3).

To determine if enhanced CDC-exosome uptake by cardiomyocytes was specific to exosomal CMP expression (and not over-expression of Lamp2b) we pre-incubated mCMs with a synthetic analog of the CMP peptide to saturate any potential receptors on the cardiomyocyte surface. Addition of the synthetic peptide prior to CMP-targeted exosomes reduced exosomal uptake to levels similar to that of non-targeted CDC-exos, demonstrating enhanced CM uptake of our targeted exosomes was specific to the CMP peptide in our cloned fusion protein (Fig. 4).

We also examined the effect of exosomal CMP-targeting on *in vitro* cardiomyocyte cell death. Consistent with our prior nSMase2 and Scr CDC coculture results, our data demonstrated significant reductions in apoptosis when cardiomyocytes were treated with CDC-derived exosomes. We further hypothesized that enhanced cardiomyocyte-specific recognition and uptake of CMP-targeted CDC-exosomes would result in an additive reduction in cardiomyocyte apoptosis. We found that pre-treatment of mCMs with CMP-targeted exosomes lead to a larger relative reduction in cardiomyocyte apoptosis (48%) (in relation to the peptide plus vehicle control) than CDC-exosome treatment alone (30%) (Fig. 5). Similar to the effect seen following pre-incubation with synthetic CMP peptide on enhanced cardiomyocyte uptake of CMP-exosomes, we found that pre-treatment with the synthetic peptide returned the levels of CM apoptosis to that seen with control exosomes alone.

To investigate cardiac retention of CMP-targeted CDC-exosomes as compared with control CDC-exosomes *in vivo*, we performed intramyocardial injections with both exosome populations. CMP-targeted CDC-exosomes demonstrated enhanced retention in the heart at 2 hours when compared with non-targeted CDC-exosomes (Fig. 6a,b). The biodistribution of targeted and non-targeted CDC-exosomes were not statistically different in any organ except for the heart, where we saw improved cardiac retention by a 4.3-fold change, further establishing the cardiac specificity of our genetically engineered CDC-exosomes.

**Figure 7:**
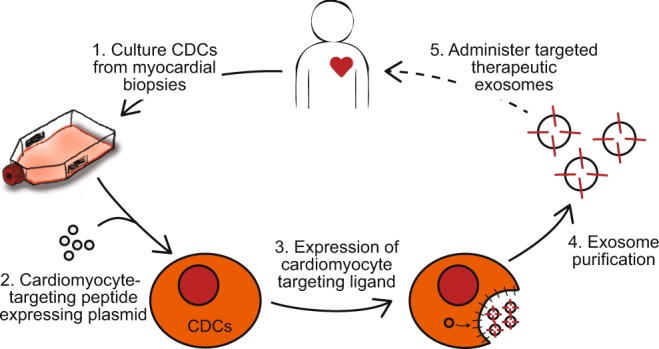
Schematic diagram demonstrates the methods for development of CDC-derived cardiomyocyte targeted exosomes and their future therapeutic potential.

Our aim in this study was to engineer CDC-exosomes to target cardiomyocytes and increase exosome retention in the heart via expression of a cardiomyocyte-specific binding peptide. By improving exosome targeting to the heart, we would anticipate increased therapeutic efficacy and decreased off target side effects. Current studies are underway to identify cardioprotective exosomal miRNAs and proteins. Combining exosomal targeting strategies with manipulation of custom cargo may result in a synergistic therapeutic effect and allow for the development of disease-specific and patient-specific therapies. In addition to the use of cell-specific peptides, additional surface protein modification can 1) aid in directed delivery (cell-specific receptors/ligands, antibodies/nanobodies for specific markers), 2) reduce exosome clearance from the circulation (blockade of SR-A), 3) enhance cytosolic delivery of exosomes (pH-sensitive surface peptides), and 4) potentiate exosome stability and delivery efficiency (glycosylation of peptides of the exosomal protein surface). A combination of methods will likely enhance the therapeutic efficacy of exosomes for use following an ischemic injury, where the therapeutic window of opportunity is limited. Future studies will exploit the peptide sequence homology with swine and are aimed at further elucidating the therapeutic effects of cardiomyocyte exosome targeting in pre-clinical large animal models of ischemic and non-ischemic heart disease with the long-term goal of bench-to-bedside translation (Fig. 7).

## Methods

All animal experiments were performed according to protocols approved by the University at Buffalo Institutional Animal Care and Use Committee. Human cardiosphere-derived cells and cardiac fibroblasts were obtained from patients who consented to tissue use under protocols approved by the University at Buffalo Research Subjects Institutional Review Board. Methods were carried out in accordance with the relevant guidelines and regulations.

### Cell culture

#### Cardiosphere-derived cells

Human cardiosphere-derived cells were generated as previously described under approved Institutional Review Board protocols with informed consent obtained from all participants^7^. Briefly, endomyocardial biopsies were minced into small fragments, washed, and digested with type IV collagenase for 60 minutes at 37 °C. Explants were cultured on 20 μg/ml fibronectin-coated dishes. A layer of stromal-like cells and population of small, round, phase-bright cells migrated out to surround the explants. Once confluent, the cells surrounding the explants were harvested by gentle enzymatic digestion. These cardiosphere-forming cells were seeded at ~1 × 10^5^ cells/mL on low attachment dishes in cardiosphere medium (20% heat-inactivated fetal calf serum, pen/strep 100 μg/ml, 2 mmol/L L-glutamine, and 0.1 mmol/L 2-mercaptoethanol in Iscove’s modified Dulbecco medium). Following 4–6 days in suspension culture, cardiospheres were harvested and plated on fibronectin-coated flasks forming monolayers of cardiosphere-derived cells. CDCs were characterized by flow cytometry and immunohistochemistry as previously described^7^.

#### Cardiomyocytes

Primary mouse cardiomyocytes were harvested and grown as previously described^7^. Briefly, day 1–3 neonatal mice were euthanized by cervical dislocation and their hearts dissected. Tissue digestion was performed using the Pierce Primary Cardiomyocyte Isolation Kit (ThermoScientific). For each experiment, dissociated cardiomyocytes were pooled from 5–10 neonatal mouse hearts (total yield approximated 6–12 × 10^6^ cells). Isolation and plating of cardiomyocytes was accomplished within two hours of animal euthanization to obtain high cell yield and reproducible viability. Cells were plated at 5.0 × 10^5^ per 24 well. Cardiomyocyte purity was over 80% at one week with strong synchronous contractions of cells in the wells visualized by phase contrast microscopy after 3 days in culture.

#### Cardiac fibroblasts

Cardiac fibroblasts were obtained from right atrial appendage endomycoardial tissue biopsies under approved Institutional Review Board protocols as described above. Cells were isolated and cultured as previously described^7^.

#### Cell lines

C2C12 mouse myoblast cells and HEK 293T cells were cultured in DMEM (Gibco) supplemented with 10% FBS and 1% pen/strep. HUVECs were cultured on collagen-coated plates in MesoEndo Cell Growth Medium (Sigma-Aldrich). All cells were incubated at 37 °C in 5% CO_2_.

### Plasmid cloning

We modified a previously characterized protocol for displaying epitopes on the external exosome surface^11^. An expanded step-by-step section on cloning methodology can be found in the Supplementary Methods. Briefly, Lamp2b was cloned from the C2C12 mouse myoblast cell line with primers containing XhoI and BspEI restriction sites to enable subsequent ligation of the targeting peptide. The Lamp2b insert was then ligated into pmEGFP-C1 (Addgene) backbone with the unique restriction sites NheI and BamHI, removing eGFP in the process. Primers encoding a cardiomyocyte-specific peptide^10^ were used to introduce the targeting moiety between XhoI and BspEI restriction sites in Lamp2b. The complete construct was then cloned into pLenti-GIII-C MV-GFP-2A-Puro to enable lentivirus particle production for eventual transduction and constitutive expression in CDCs. The final plasmid, consisting of Lamp2b and a cardiomyocyte targeting peptide within a lentiviral vector backbone, is referred to as pLenti-CMP for simplicity. Gel electrophoresis was performed and imaged using a ChemiDoc^TM^ XRS + Imaging System7 (Bio-Rad Laboratories). Sanger sequencing of Lamp2b, pCMP, and pLV-CMP plasmids was conducted at Roswell Park Cancer Institute (Buffalo, New York) using Lamp5F and Lamp3R primers. All primers used for RT-PCR and cloning are listed in Table 1.

**Table 1:** Primers used for RT-PCR, cloning and quantitative PCR.

Primer	Sequence (5′->3′)
**Lamp2b RT-PCR**	
Lamp5-F	ATCCGCTAGCGGTCGCCACCATGTGCCTCTCTCCGGTT
Lamp5-R	GTCACTCGAGCATAAAGGCAAGTACCCTTTGAA
Lamp3-F	GTCACTCGAGGTCACATCCGGAGGTGCAGAATGGGAGATGAATTTCA
Lamp3-R	ATCCGGATCCTTAGTGTTACAGAGTCTGATATCC
**Targeting Peptide**	
CMP-F	TCGATGGCTGAGCGAAGCGGGCCCGGTGGTGACCGTGCGCGCGCTGCGCGGCACCGGCAGCTGGT
CMP-R	CCGGACCAGCTGCCGGTGCCGCGCAGCGCGCGCACGGTCACCACCGGGCCCGCTTCGCTCAGCCA
**Quantitative PCR**	
Gapdh-F	ACCACAGTCCATGCCATCAC
Gapdh-R	CACCACCCTGTTGCTGTAGCC
CMPLamp2b-F	GTGTGTCGCCTTGTCAGGTA
CMPLamp2b-R	ATGGCTGAGCGAAGCGG

### Quantitative real time PCR

RNA was extracted from cells using an E.Z.N.A Total RNA Kit I (Omega Bio-tek) and cDNA synthesized with SuperScript III reverse transcriptase (Thermo Fisher Scientific). Primers used for SYBR Green-based PCR are provided in Table 1. Samples were run in triplicate and gene expression calculated by ΔΔC_t_ analysis using 18S as a reference. Statistical significance was tested on log_2_-transformed data using ANOVA.

### Lentivirus generation and creation of lamp2b-CMP CDC cell line

Lentivirus was prepared as previously described^7^. Briefly, Lamp2b-CMP lentivirus was generated by triple transfection of 293 T cells with the lentiviral backbone pLenti-CMP and packaging plasmids pLP/VSVG (Invitrogen) and psPAX2 (AddGene) using Fugene HD (Promega). Viral supernatant was collected at 48 and 72 h and yielded a titer of at least 10^6^ GFP-transducing U/ml.

### Exosome isolation and characterization

Exosomes were harvested from CDCs grown in media containing exosome-free serum as previously described^7^ and characterized by surface protein expression, morphology, size, and surface charge.

#### Exosome surface protein

50 ug of exosomal protein was used for antibody detection of known exosomal markers CD63, CD81, ALIX, FLOT1, ICAM1, EpCam, ANXA5, and TSG101 as well as markers of cellular contamination (Exo-Check Exosome Antibody Array, System Biosciences).

#### Cryo-TEM

Cryo-TEM was performed as previously described^28^. Briefly, a FEI Vitrobot (Mark IV) plunge freezer, set at room temperature and approximately 95% humidity, was used to prepare vitrified cryo-TEM specimens from the aqueous samples. Approximately 2.5 µL of a 1 mg/ml exosome solution was applied to a TEM grid coated with lacey carbon film. After blotting using two filter papers, the grid was plunge-frozen in liquid ethane^29^. The vitrified specimen was mounted onto a Gatan 626.DH cryo-TEM holder and transferred into a FEI Tecnai F20 TEM equipped with a Gatan twin blade retractable anti-contaminator. The cryo-TEM observation was carried out at approximately −174 °C.

#### Nanoparticle tracking analysis

Nanoparticle tracking analysis (NTA) was performed as previously described (inc ref.^7^). NTA data was collected for 60 seconds with 30 ms shutter and manual gain adjustments (screen gain: 6, detection threshold: 6, minimum particle size: 30 nm). A temperature probe was used to verify that all measurements were taken at room temperature.

#### Surface charge

Exosome zeta potential was analyzed with triplicate samples diluted in H_2_0 using the SZ-100 (HORIBA Scientific).

### *In vitro* assessment of exosome uptake

Exosomes were isolated from conditioned media of CDCs and resuspended in 500 ul of 1x PBS. Exosomes were labeled with Exo-Red (Acridine Orange chemistry) (System Biosciences) as previously described^7^. Target cells were incubated with labeled exosomes and their uptake was assessed by fluorescence microscopy (Invitrogen EVOS FL Auto 2) at 2 and 24 hours at both 10x and 20x magnification. Parameters were controlled between image acquisition steps by creating an automatic scanning protocol. Fluorescent-labeled exosome uptake was quantified with ImageJ using the Color Threshold technique adapted from Jensen^30^ with slight modifications. Briefly, a color threshold was applied to each image with the following parameters: Hue: 0–255, Saturation: 0–255, Brightness: 31–255, Threshold method: Triangle. After thresholding, the Integrated Density (pixel area × mean gray value) was calculated for each image. Images were adjusted and scaled identically, and no scaling units were added post-image acquisition.

### CMP-targeted exosome specificity assay

Neonatal mouse cardiomyocytes were plated at 4.0 × 10^4^ cells per well in a 96-well plate and grown for 4 days in cardiomyocyte media (ThermoScientific). 30 minutes prior to exosome treatment, cardiomyocytes were pre-treated with either 10 μM of CMP peptide (WLSEAGPVVTVRALRGTGSW, Sigma) or DMSO (vehicle carrier). 1.2 µg of Exo-Red labeled CDC-exosomes or CMP-targeted CDC-exosomes were added to each well and uptake assessed at 2 and 24 hours by fluorescent microscopy (Invitrogen EVOS FL Auto 2, 20x magnification). Image acquisition and analysis was controlled by using an automatic scanning protocol and the Color Threshold technique described above.

### Primary cardiomyocyte apoptosis quantification by TUNEL

Neonatal mouse cardiomyocytes were plated and cultured as described above. 30 minutes prior to exosome treatment, cardiomyocytes were pre-treated with either 10 µM of CMP peptide or vehicle control (10 µM DMSO). Cardiomyocytes were incubated with 1.2 ug of CDC-exosomes or CMP-targeted CDC-exosomes for 24 hours prior to fixation with 4% PFA. Apoptosis was assessed by terminal deoxynucleotidyl transferase-mediated dUTP nick end-labeling (TUNEL) following the manufacturer’s protocol (Click-iT™ TUNEL Alexa Fluor™ 488, Invitrogen). Cells were counterstained with TroponinI (TnI) and DAPI for quantification of apoptotic cardiomyocytes. Only those cells that stained positive for TnI and DAPI were counted as cardiomyocytes, and only cells that stained positive for TnI, DAPI, and TUNEL were counted as apoptotic cardiomyocytes.

### *In vivo* bio-distribution of exosomes

Exosome pellets were suspended in 500 μL of 1x PBS and combined with 50 μL of DiR (near IR fluorescent, lipophilic carbocyanine, Thermo Fisher). The mixture was inverted several times and incubated at 37 °C for 10 minutes. 100 μL of ExoQuick-TC (System Biosciences) was added, inverted to mix and placed on ice for 30 minutes. The solution was centrifuged at 14,000xg for 3 minutes and the supernatant containing free dye aspirated and discarded. Exosomes were resuspended in PBS for subsequent *in vivo* use.

Intravenous injections were performed using a retro-orbital route as previously described^4^. C57BL/6 J adult mice were anesthetized with isoflurane, placed in a left lateral recumbent position, and gentle pressure applied to the skin dorsal and ventral to the eye so as not to impede blood flow to the ventral cervical vessels or injure the trachea. A Hamilton syringe with a sterile 30.5-gauge beveled needed was loaded with 10 μl of DiR-labeled exosomes (400 ng/μl) or 100 μl of PBS and advanced bevel down at an angle of approximately 30° into the medial canthus. The injectate was slowly and smoothly delivered over a period of 10 seconds then slowly and smoothly withdrawn.

Intramyocardial injections were performed under direct visualization via a left thoracotomy. Anesthesia was induced in adult C57BL/6J mice with a mixture of ketamine (100 mg/ml) and xylazine (20 mg/ml). A midline ventral cervical skin incision was performed and the muscles overlying the larynx and trachea were bluntly dissected and retracted to allow visualization of the intubation device through the exposed trachea. To intubate the mouse, the tongue was slightly retracted, and a 20-gauge 1” smooth needle was inserted through the mouth and larynx and into the trachea with care taken not to puncture the trachea or other structures in the pharyngeal region. The needle was advanced 8 to 10 mm from the larynx and taped in place to prevent dislodgment. Mice were ventilated with room air supplemented with oxygen (1 L/min) at a rate of 105 breaths/min and a tidal volume of 10.3 μL/g using a mouse ventilator (Inspira asv, Harvard Apparatus, Germany)^31^. The left chest was shaved, and a 1.5 cm skin incision made along the mid-axillary line. The left pectoralis major muscle was bluntly dissociated exposing the ribs. The muscle layers were retracted, and a left thoracotomy performed between the third and fourth ribs to visualize the anterior surface of the heart and left lung. A Hamilton syringe loaded with either 10 μl of DiR-labeled exosomes (400 ng/μl) or 10 μl of PBS and a 30.5-gauge sterile beveled needle was introduced into the left ventricle so the bevel was just under the myocardium. The solution in the syringe was slowly injected watching for local tissue blanching indicating myocardial vs. LV cavity delivery. The syringe was held in place for an additional 3–5 seconds then slowly withdrawn. If there was any bleeding, a cotton-tipped applicator was gently pressed onto the needle insertion site until the bleeding stopped. Using 6–0 polypropylene suture, the intercostal space was closed in an x-mattress pattern, followed by closure of the muscle layer in an interrupted suture pattern, and skin layers in a running mattress pattern. Mice received post-operative analgesia with 0.05 mg/kg SQ Buprenorphine and were recovered on a heating pad. Two hours following intramyocardial injection, mice were euthanized by inhaled isoflurane overdose and subsequent cervical dislocation, and organs harvested and placed on ice in PBS in the dark for subsequent assessment of biofluorescence (IVIS Spectrum, Roswell Park Comprehensive Cancer Center). Images were taken and analyzed with Living Image using the following specifications: Excitation −745 nm, Emission −820 nm, FOV −13.4 cm, Auto Shutter Time.

## Supplementary information

Supplementary Information File

## Data Availability

All data generated or analyzed during this study are included in this published article (and its Supplementary Information files).
