# Supplementary material for: Exosomes Engineered to Express a Cardiomyocyte Binding Peptide Demonstrate Improved Cardiac Retention in Vivo
Source: Sci Rep. 2019 Jul 11;9:10041. doi: 10.1038/s41598-019-46407-1 (PMC6624248; doi:10.1038/s41598-019-46407-1)
Supplement: Supplementary file 1 — Supplementary Information File [file 41598_2019_46407_MOESM1_ESM.pdf]

Supplementary Materials

Exosomes Engineered to Express a Cardiomyocyte Binding Peptide Demonstrate Improved Cardiac Retention *in Vivo*

Kyle I. Mentkowski[1,2] and Jennifer K. Lang[1,2,3*]

[1]Department of Medicine, Division of Cardiology, Jacobs School of Medicine and Biomedical Sciences, Buffalo, N.Y., 14203, United States of America

[2]Department of Biomedical Engineering, University at Buffalo, Buffalo, N.Y., 14260, United States of America

[3]VA WNY Healthcare System, Buffalo N.Y., 14215, United States of America

Outline

Expanded cloning methodology (including Supplementary Figs. S1-S4)...…………2-8

Supplementary Fig. S5……………………………………………………………………9

Supplementary Fig. S6……………………………………………………………………10

Expanded cloning methodology

1. RNA was extracted from C2C12 cells using the E.Z.N.A. Total RNA Kit I (Omega Bio-tek, Norcross, GA, USA) protocol. Synthesis of cDNA was performed using the Super Script® III First-Strand Synthesis System using Lamp3R as the gene-specific primer (Thermo Fischer Scientific Inc.). Accuzyme DNA Polymerase (Bioline, MA, USA) was utilized to amplify Lamp2b as two separate fragments (Lamp5F + Lamp5R, Lamp3F + Lamp3R). PCR reaction was performed using volumes/concentrations of reagents as listed in the following tables.

Components	Amount
**Accuzyme Buffer (10X)**	5 µL
**Lamp5 Primers (10 µM)**	2.5 µL (F and R)
**dNTP Mix (10 mM)**	1 µL
**cDNA template**	1 µg
**Accuzyme (2.5 U/µL)**	1 µL
**Water (ddH$_2$O)**	Up to 50 µL

Components	Amount
**Accuzyme Buffer (10X)**	5 µL
**Lamp3 Primers (10 µM)**	2.5 µL (F and R)
**dNTP Mix (10 mM)**	1 µL
**cDNA template**	1 µg
**Accuzyme (2.5 U/µL)**	1 µL
**Water (ddH$_2$O)**	Up to 50 µL

2. An Applied Biosystems GeneAmp PCR System 9700 (Thermo Fischer Scientific Inc.) was utilized for the PCR reaction and was set according to the conditions listed in the following table.

Cycle Number	Step	Temperature	Time
**1**	Denature	95°C	2 Minutes
**2a-39a**	Denature	95°C	20 Seconds
**2b-39b**	Anneal	54°C	20 Seconds
**2c-39c**	Extend	68°C	2 Minutes
**40**	Hold	4°C	Until Removal

3. The creation of Lamp2b relied on components that were prepared in three separate digestion reactions. Two of the reactions were set up according to the following table.

Components	Reaction 1	Reaction 2
**NEB Buffer 2 (10X)**	5 µL	
**NEB Buffer 3 (10X)**		5 µL
**LAMP5 PCR Product**	25 µL	
**LAMP3 PCR Product**		25 µL
**NheI (10 U/µL)**	1 µL	
**BamHI (20 U/µL)**		1 µL
**XhoI (20 U/µL)**	1 µL	1 µL
**Water (ddH$_2$O)**	Up to 50 µL	Up to 50 µL

4. These components were incubated at 37°C for 2 hours to create compatible restriction ends for ligation of the PCR products into pmEGFP-C1. Due to the incompatibility of NheI and BamHI in double digestion reactions, a third sequential digestion reaction was required to remove the EGFP from pmEGFP-C1. This reaction was set up according to the following table.

Components	Reaction 3
**NEB Buffer 2 (10X)**	5 µL
**pmEGFP-C1 plasmid**	1 µg
**NheI (10 U/µL)**	1 µL
**Water (ddH_2O)**	Up to 50 µL

5. Because NheI requires a lower salt concentration, the pmEGFP-C1 plasmid was first incubated with NheI at 37°C for 1 hour to digest the 5' end of pmEGFP-C1. BamHI was then added to digest the 3' end of pmEGFP-C1. The salt concentration was adjusted to that of NEB Buffer 3 by adding 2.5 µL of 1M NaCl.

Components	Reaction 3
**BamHI (20 U/µL)**	1 µL
**NaCl (1M)**	Adjust to NEB Buffer 3 Salt Conc.

6. To prevent reassembly of pmEGFP-C1, the sticky ends were dephosphorylated by the addition of 1 µL of calf intestinal phosphatase (CIP). The mixture was incubated for 30 minutes at 37°C. A ligation reaction was set up to create pLamp2b from the pieces that had been prepared. The reaction was prepared according to the following table.

Components	Amount
**T4 DNA ligase buffer (10X)**	1 µL
**LAMP5 digested product**	6 ng
**LAMP3 digested product**	30 ng
**pmEGFP-C1 digested product**	40 ng
**T4 DNA ligase (400 U/µL)**	1 µL
**Water (ddH_2O)**	Up to 10 µL

7. The reaction was carried out overnight at 16°C. The pLamp2b plasmid resulting from this ligation reaction causes transfected cells to produce exosomes that express Lamp2b, the N-terminus of which resides on the surface of exosomes (Supplementary Fig. S1)

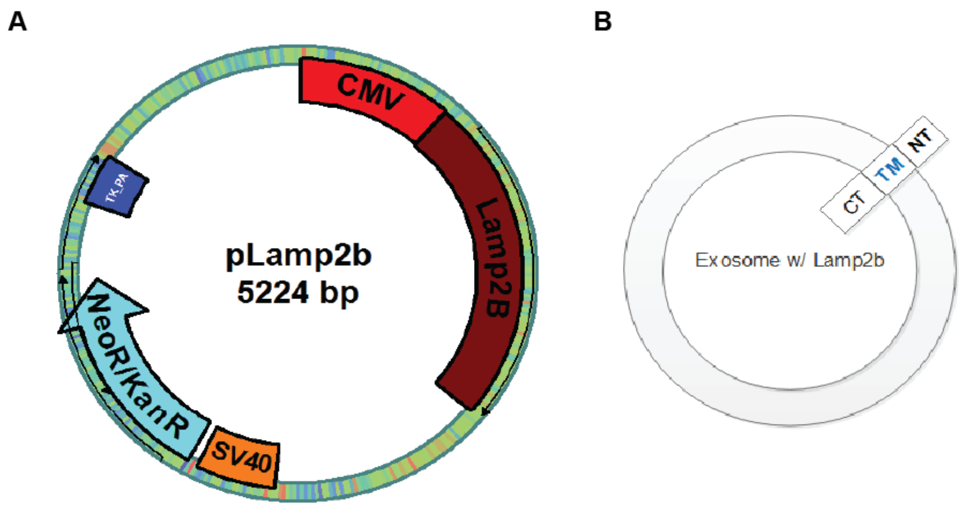

Supplementary Figure S1. (A) The restriction sites Nhe1 and BamH1 were used to ligate a sequence encoding for the Lamp2b transmembrane protein into pmEGFP-C1. Image was created using pDraw32. **(B)** After transfection with the pLamp2b plasmid, cells produce exosomes that express Lamp2b transmembrane proteins. These proteins allow for the addition of a targeting peptide. CT: C-Terminus. TM: Trans-Membrane. NT: N-Terminus. Image was created using Edraw Max.

8. To ensure that CMP primers contained a 5’ phosphate, the following phosphorylation reaction was set up and run in a thermocycler with the following cycle specifications: 30 minutes at 37°C, 95°C for 5 minutes, and cooled by 0.5°C every 10 seconds until reaching a final temperature of 25°C.

Components	Amount
**T4 DNA ligase buffer (10X)**	5 µL
**Forward CMP Primer (100 µM)**	1 µL
**Reverse CMP Primer (100 µM)**	1 µL
**T4 polynucleotide kinase (10,000 U/mL)**	1 µL
**Water (ddH$_2$O)**	Up to 50 µL

9. The phosphorylated CMP primers were flanked by the restriction enzymes XhoI and BspEI, which allowed for the controlled integration of the insert into the pLamp2b backbone at the correct location in the Lamp2b sequence. This double digestion reaction was prepared according to the following table:

Components	Amount
**NEB Buffer 3 (10X)**	5 µL
**pLamp2b**	1 µg
**XhoI (20 U/µL)**	1 µL
**BspEI (10 U/µL)**	1 µL
**Water (ddH$_2$O)**	Up to 50 µL

10. This reaction was incubated for 2 hours at 37°C before 1 µL of calf intestinal phosphatase (CIP) was added. The mixture was then incubated for 30 minutes at 37°C, at which point, the double digestion reaction was complete. A subsequent ligation reaction was set up to insert the CMP primer into the digested pLamp2b vector. The reaction was prepared according to the following table.

Components	Amount
**T4 DNA ligase buffer (10X)**	1 µL
**Digested pLamp2b**	60 ng
**Annealed CMP Primer**	1 µL
**T4 DNA ligase (400 U/mL)**	1 µL
**Water (ddH_2O)**	Up to 10 µL

11. This reaction occurred overnight in a thermocycler set to 16°C. The product of this reaction was a pCMP plasmid that would cause the exosomes of transfected cells to express Lamp2b proteins with a cardiomyocyte-specific targeting peptide on their surface (Supplementary Fig. S2).

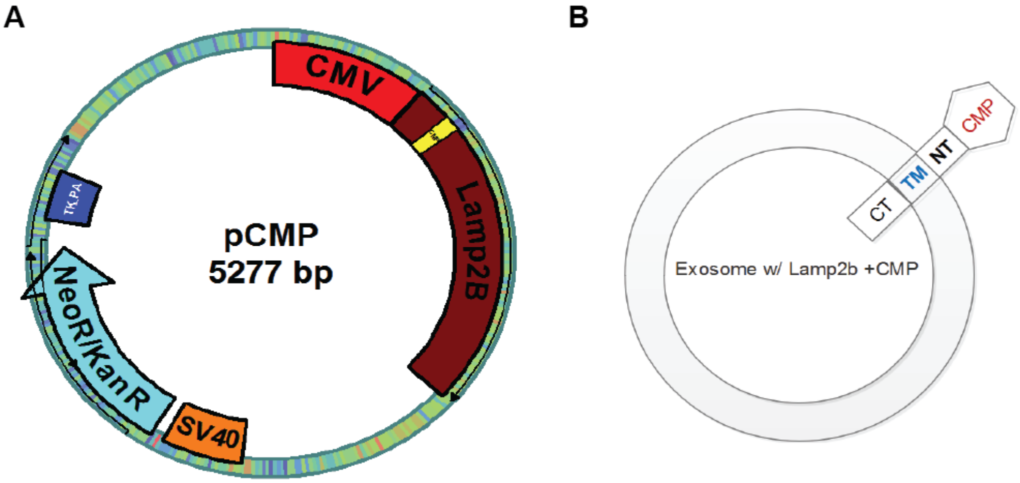

Supplementary Figure S2. (A) A ligation reaction was performed such that a sequence coding for a cardiomyocyte targeting peptide was introduced within the Lamp2b sequence of the plasmid. Image was created using pDraw32. **(B)** The sequence for the cardiomyocyte-specific targeting peptide was ligated into the Lamp2b sequence in a location that caused the cardiomyocyte peptide (CMP) to be expressed on the outside surface of the exosome. Image was created using Edraw Max.

12. The resulting plasmid pCMP was transformed into *E. coli*. Plasmids were then isolated from bacterial cells using the previously described protocol for the E.Z.N.A. Endo-Free Plasmid DNA Mini Kit (Omega Bio-tek, Norcross, GA, USA).

13. To increase transfection efficiency, a final digestion/ligation reaction was performed to transfer the insert of interest from the pCMP plasmid into a lentiviral vector backbone. The following digestions were performed.

Components	Amount
**NEB Buffer 2 (10X)**	5 μL
**pLenti backbone**	1 μg
**NheI (10 U/μL)**	1 μL
**Water (ddH_2O)**	Up to 50 μL

Components	Amount
**NEB Buffer 2 (10X)**	5 μL
**pCMP plasmid**	1 μg
**NheI (10 U/μL)**	1 μL
**Water (ddH_2O)**	Up to 50 μL

14. For insertion of the sequence, the pLenti-GIII-CMV-GFP-2A-Puro vector (Applied Biological Materials Inc.) was chosen. This vector had NheI and BamHI restriction sites, the same sites at the 5' and 3' ends that flanked the insert sequence. The 5' ends of the pCMP and pLenti plasmids were cleaved through a digestion with NheI for 1 hour at 37°C. To cleave the 3' ends, BamHI was added to each reaction along with 2.5 μL of 1 M NaCl to reach the proper salt concentration for the optimal function of BamHI. Each reaction was incubated for 1 hour at 37°C. The reactions were prepared according to the following table.

Components	Amount
**BamHI (20 U/μL)**	1 μL
**NaCl (1 M)**	Adjust to NEB Buffer 3 Salt Conc.

15. To avoid reassembly of the digested plasmids, calf intestinal phosphatase was added and the mixture was incubated for 30 minutes at 37°C. Gel electrophoresis was performed as previously described to analyze the size of the digestion products and to isolate the pLenti vector backbone and pCMP insert. To perform the gel electrophoresis, 40 μL of each digestion product was loaded into an agarose gel. After electrophoresis, the gels showed that there were two bands for pLenti-GIII-CMV-GFP-2A-Puro (backbone and cassette insert) and two bands for pCMP (backbone and insert). The QIAquick™ Gel Extraction Kit was used to isolate the pLenti-GIII-CMV-GFP-2A-Puro backbone and the pCMP insert from excised gel according to the manufacturer's protocol. A final ligation reaction was prepared in an Eppendorf tube according to the following table.

Components	Amount
**T4 DNA ligase buffer (10X)**	2 μL
**Lamp2b + CMP insert**	22 ng
**pLenti backbone**	50 ng
**T4 DNA ligase (400 U/μL)**	1 μL
**Water (ddH_2O)**	Up to 20 μL

16. The NEBioCalculator was used to determine the proper concentrations for the ligation reaction to occur. After incubating overnight at 16°C, the ligated product was transformed into One Shot® TOP10 Chemically Competent *E. coli* (ThermoFischer). The E.Z.N.A. Endo-Free Plasmid DNA Mini Kit was then used according to the previously described protocol to isolate the amplified pLenti-GIII-CMV-GFP-2A-Puro-CMP (LV-CMP) (Supplementary Fig. S3).

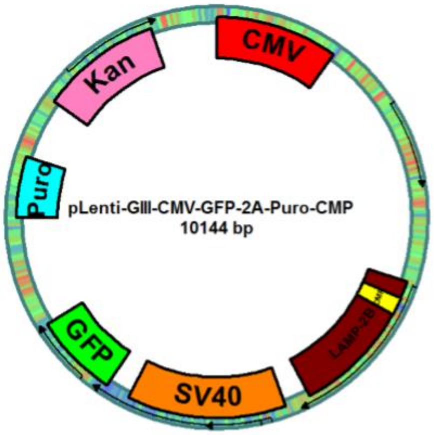

Supplementary Figure S3. The transfer vector shown above, referred to as LV-CMP, contains the ligated Lamp2b + CMP insert. LV-CMP, VSV-G, and psPAX2 were used to create a lentivirus with optimal transfection efficiency in CDCs. Vector backbone purchased from Applied Biological Materials Inc. Image created using pDraw32.

17. Plasmids and inserts were additional characterized throughout the protocol by sequential restriction digests (Supplementary Figure S4).

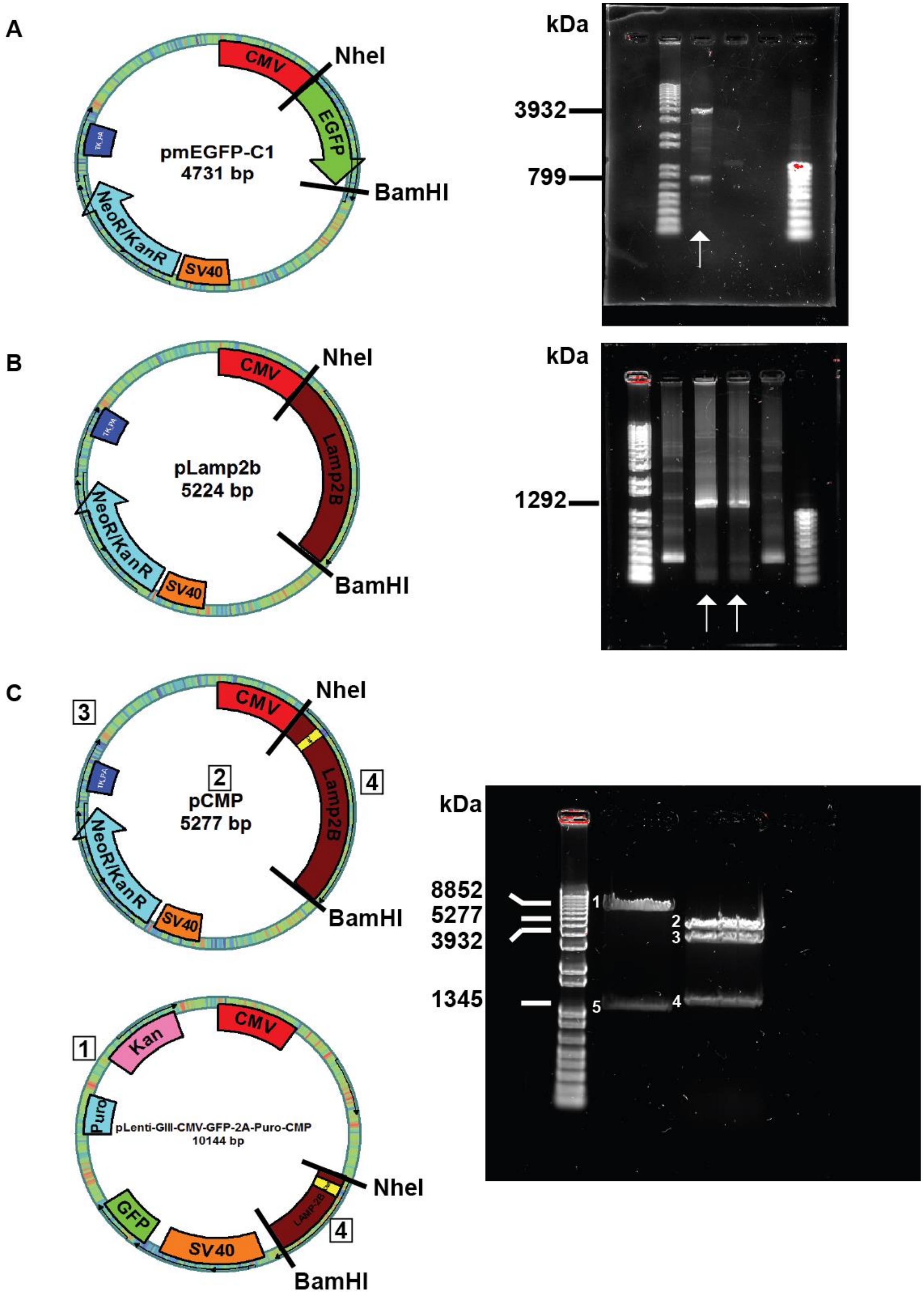
A
CMV
NheI
EGFP
BamHI
TK pA
pmEGFP-C1
4731 bp
NeoR/KanR
SV40
kDa
3932
799
B
CMV
NheI
TK pA
pLamp2b
5224 bp
Lamp2B
NeoR/KanR
SV40
BamHI
kDa
1292
C
3
CMV
NheI
2
pCMP
5277 bp
4
Lamp2B
TK pA
NeoR/KanR
SV40
BamHI
1
Kan
CMV
Puro
pLenti-GIII-CMV-GFP-2A-Puro-CMP
10144 bp
NheI
GFP
SV40
LAMP-2B
4
BamHI
kDa
8852
5277
3932
1345
1
2
3
4
5

Supplementary Figure S4: Summary of cloning plasmids and insert validation by restriction digest. A. pmEGFP-C1 (AddGene) was digested with NheI and BamHI, removing eGFP and creating space for the ligation of Lamp2b. Gel electrophoresis results highlight the size of the pmEGFP-C1 backbone and eGFP insert (arrow represents lane of interest). **B.** Lamp2b was amplified from C2C12 cDNA (Supplementary Methods) and ligated into the pmEGFP-C1 backbone, generating pLamp2b (arrows represent clones of interest). **C.** pLamp2b was digested with XhoI and BspEI and the CMP amplified product was ligated into pLamp2b to create pCMP. This plasmid, along with pLenti-GIII-CMV-GFP-2A-Puro (a lentiviral transfer plasmid), was subsequently digested with NheI and BamHI to remove the entire CMP + Lamp2b sequence in order to exchange this region for the cassette contained within the lentiviral transfer plasmid. Gel electrophoresis results: 1. Transfer Plasmid backbone, 2. pCMP plasmid, 3. pCMP backbone, 4. CMP + Lamp2b insert, 5. Endogenous transfer plasmid cassette.

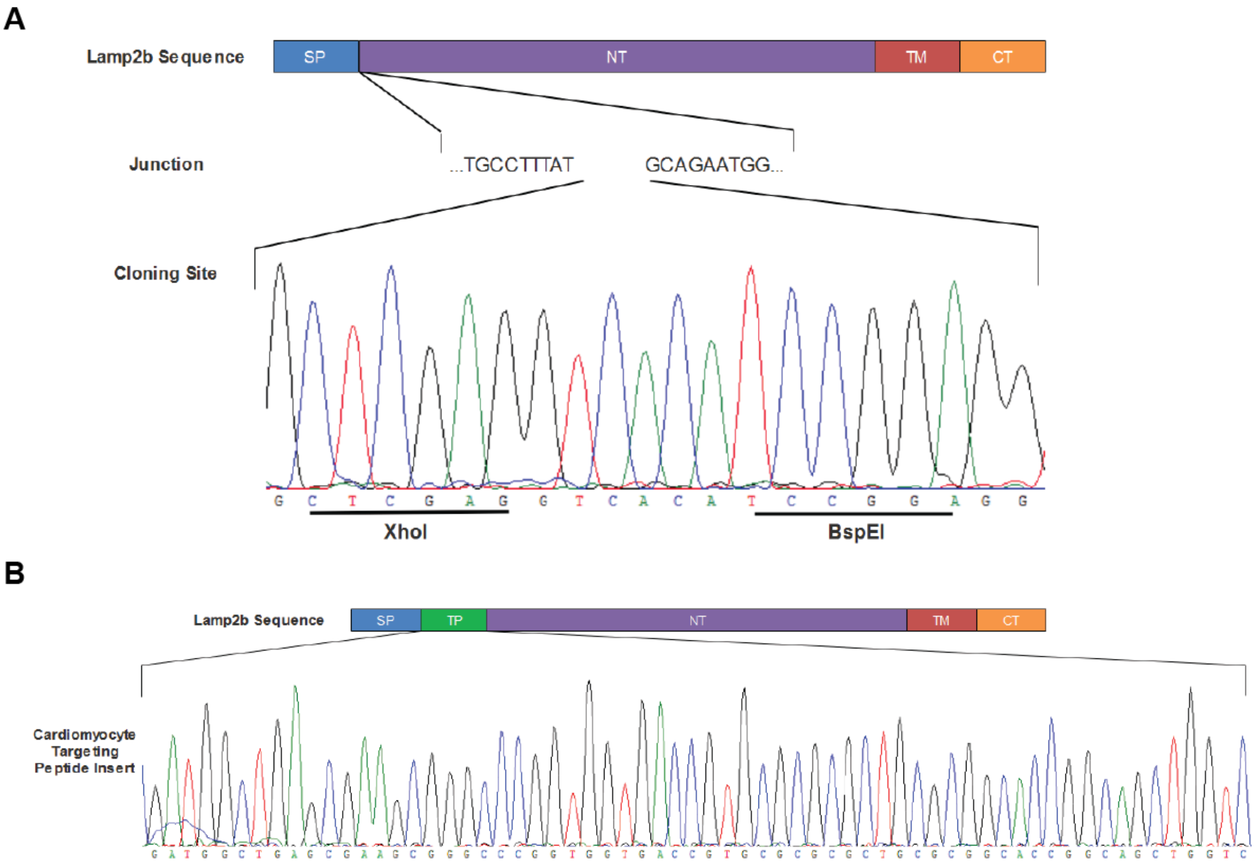

Supplementary Figure S5: Schematic of Lamp2b cloning and sequencing results. A. Location within the native Lamp2b gene, between restriction sites XhoI and BspEI, where a potential targeting peptide can be ligated. SP: Signal Peptide, NT: N-terminus, TM: Transmembrane Region, CT: C-terminus. pLamp2b was sequenced to verify proper orientation and appearance of XhoI and BspEI. **B**. Sequencing results of pLenti-GIII-CMV-GFP-2A-Puro-CMP (LV-CMP) confirmed ligation of CMP between XhoI and BspEI in the Lamp2b gene. TP: Targeting Peptide.

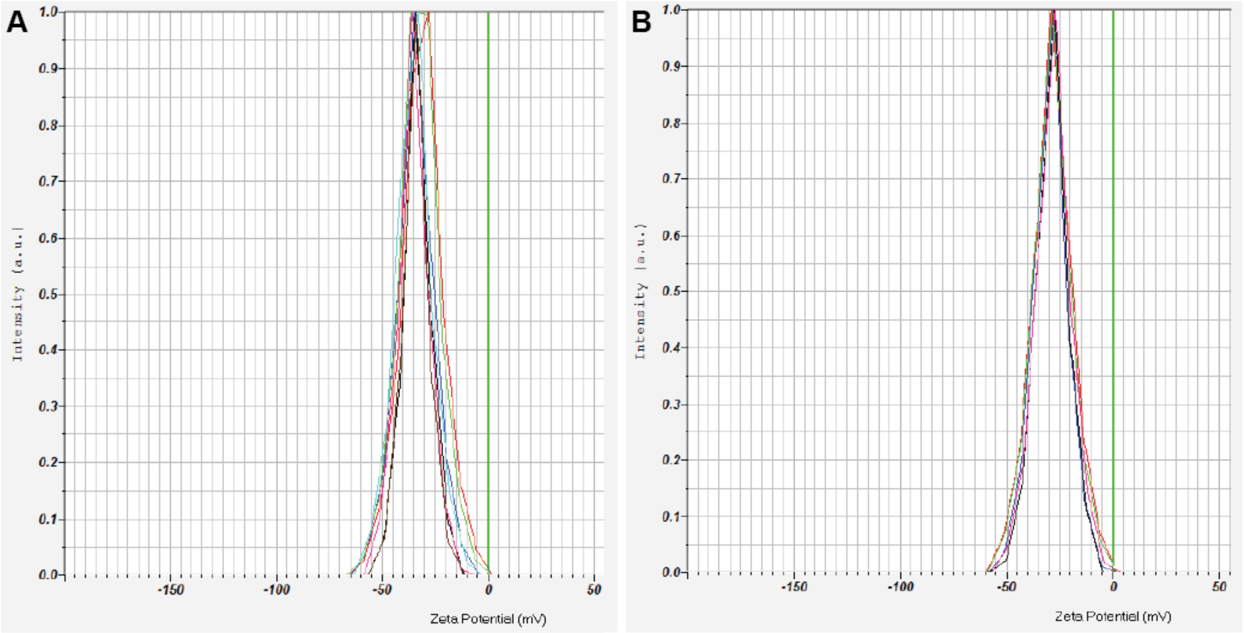

Supplementary Figure S6. Zeta Potential. Exosome zeta potential analysis revealed similar surface charges between **A.** control CDC-exosomes and **B.** CMP-targeted exosomes. Each peak represents a single measurement of either CMP-targeted or control CDC-exosomes. Both samples were run in triplicate to verify accuracy.
